# Development of a tool to assess oral health-related quality of life in patients hospitalised in critical care

**DOI:** 10.1007/s11136-019-02335-1

**Published:** 2019-10-26

**Authors:** Federico Moreno Sancho, Georgios Tsakos, David Brealey, David Boniface, Ian Needleman

**Affiliations:** 1 Unit of Periodontology, UCL Eastman Dental Institute, 1st Floor Levy wing, 256 Gray’s Inn Road, London, WC1X 8LD UK; 2 Department of Epidemiology and Public Health, University College London, 1 - 19 Torrington Place, London, WC1E 7HB UK; 3 Bloomsbury Institute of Intensive Care Medicine, UCL, London, UK; 4 Epidemiology and Public Health, UCL Eastman Dental Institute, University College London, 256 Gray’s Inn Road, London, WC1X 8LD UK; 5 Biostatistics, UCL Eastman Dental Institute, UCL, 256 Gray’s Inn Road, London, WC1X 8LD UK; 6 Centre for Oral Health and Performance, UCL Eastman Dental Institute, UCL, 256 Gray’s Inn Road, London, WC1X 8LD UK

**Keywords:** Oral health-related quality of life, Critical care unit, Validation, Questionnaire, Adults

## Abstract

**Aims and objectives:**

Oral health deteriorates following hospitalisation in critical care units (CCU) but there are no validated measures to assess effects on oral health-related quality of life (OHQoL). The objectives of this study were (i) to develop a tool (CCU-OHQoL) to assess OHQoL amongst patients admitted to CCU, (ii) to collect data to analyse the validity, reliability and acceptability of the CCU-OHQoL tool and (iii) to investigate patient-reported outcome measures of OHQoL in patients hospitalised in a CCU.

**Methods:**

The project included three phases: (1) the development of an initial questionnaire informed by a literature review and expert panel, (2) testing of the tool in CCU (*n *= 18) followed by semi-structured interviews to assess acceptability, face and content validity and (3) final tool modification and testing of CCU-OHQoL questionnaire to assess validity and reliability.

**Results:**

The CCU-OHQoL showed good face and content validity and was quick to administer. Cronbach’s alpha was 0.72 suggesting good internal consistency. For construct validity, the CCU-OHQoL was strongly and significantly correlated (correlation coefficients 0.71, 0.62 and 0.77, *p* < 0.01) with global OHQoL items. In the validation study, 37.8% of the participants reported a deterioration in self-reported oral health after CCU admission. Finally, 26.9% and 31% of the participants reported considerable negative impacts of oral health in their life overall and quality of life, respectively.

**Conclusions:**

The new CCU-OHQoL tool may be of use in the assessment of oral health-related quality of life in CCU patients. Deterioration of OHQoL seems to be common in CCU patients.

**Electronic supplementary material:**

The online version of this article (10.1007/s11136-019-02335-1) contains supplementary material, which is available to authorised users.

## Introduction

During the last 30 years, much emphasis has been placed on the importance of patient-reported outcome measures (PROM) in research investigating patients hospitalised in critical care [1]. It is well established that oral health status is one of the determinants of quality of life [2]. Poor oral health has been shown to negatively impact quality of life across different populations [3–8]. Therefore, it is of concern that recent evidence suggests that hospitalisation is associated with deterioration in oral health as shown by increased plaque levels, gingival bleeding and xerostomia [9, 10]. The decline in oral health during hospitalisation is of the utmost importance: this population is more vulnerable to oral disease, this deterioration may potentially affect their quality of life and poor oral health may result in a greater risk of Ventilator Associated Pneumonia (VAP) as it is reported that VAP-associated pathogens may translocate from the oral cavity into the lungs. When patients develop VAP, this infection results in an increase of mortality, length of CCU stay and cost. A systematic review that included 11 trials and 3242 patients concluded that “Oral decontamination of mechanically ventilated adults using antiseptics is associated with a lower risk of ventilator associated pneumonia” indicating that fourteen patients would need to receive this intervention to prevent one case of VAP [11]. Poor oral health might add an additional burden, affecting the patient’s comfort and limiting the ability to eat and speak in these already compromised patients [12]. Therefore, deterioration of oral health in CCU patients might constitute a public health issue, as it could reduce quality of life, with evidence indicating that it also confers an increased risk for nosocomial infections [11]. A major limiting factor to further research is the absence of a validated tool to measure oral health-related quality of life in CCU patients. Commonly used generic OHQoL questionnaires include a large number of questions which are irrelevant for the critically ill given their life circumstances, limiting their applicability in this setting. Furthermore, these questionnaires take a long time to complete and are not feasible if the patients are frail and severely compromised, as it is the case in a CCU, because of the burden placed on patients and/or healthcare givers. Therefore, the aim of this study was to develop and validate a suitable tool to assess the impact of critical care on oral health-related quality of life (OHQoL) and to investigate patient-reported outcome measures of OHQoL in patients hospitalised in a CCU.

## Materials and methods

The study was conducted in accordance with the principles of the Declaration of Helsinki (ethical approval NRES REC London - Fulham, IRAS Project ID 136965).

**Fig. 1:**
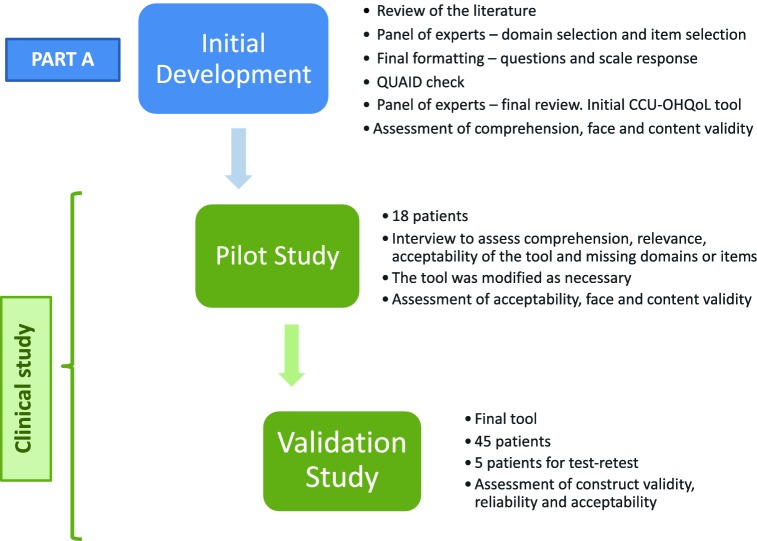
Study flow diagram

We followed a multi-stage approach similar to that described by Guyatt and co-workers [13]. The project included three phases: (a) Initial tool development, (b) Pilot study and (c) Validation study; see Fig. 1 for study flow diagram. Subjects were recruited for the study from the CCU at University College London Hospital (UCH), a mixed adult unit. Patient recruitment for Part B of the project took place from June 2014 to September 2014 and for the final validation study from December 2014 to June 2015.

### Eligibility criteria

1. Admitted to CCU for at least 48 h
2. Received level two or level three care as described by the Intensive Care Society Standards
3. Able to communicate in English to the nurse/assessor (Glasgow Coma Scale (GCS) of 15; Richmond Agitation Sedation Scale (RASS) score between 0 and 1)
4. At least 18 years of age

### Part A: initial development and creation of the initial tool

Conceptually, for the development of the questionnaire, we used the definition of OHQoL from Locker and Allen: “the impact of oral disorders on aspects of everyday life that are important to patients and persons, with those impacts being of sufficient magnitude, whether in terms of severity, frequency or duration, to affect an individual’s perception of their life overall” [14]. A literature search was performed which ascertained that no research was available in relation to OHQoL in CCU nor were there any validated tools to measure the OHQoL of patients at a CCU. We reviewed the literature to identify the most common oral health problems experienced by those hospitalised in CCU. We mapped the items and domains obtained from the literature and sought advice from a panel of experts, including consultants and nurses in critical care and experts in oral care and public health, to compare them against existing OHQoL tools validated in other settings. The domains and items were revised and re-presented to the expert panel until consensus was reached.

The comprehension of each of the items was initially assessed using the Question Understanding Aid tool (QUAID) and items modified as required [15]. As a result of this initial research, an initial CCU-OHQoL tool was created (Supplementary Files—Initial tool).

### Clinical project

#### Part B: pilot study—domain and item revision, modification of initial tool

The domain and item revision involved administering the tool in a pilot study involving 18 CCU patients before discharge. The patients assessed the interpretability, relevance and acceptability of the CCU-OHQoL. After the questionnaire was completed, the nurses conducted a short interview comprising a series of open-ended questions with regard to the validity of the domains and items of the questionnaire and to identify possibly missing items. The initial tool was modified as necessary.

#### Part C: validation study—construct validity, reliability and acceptability

The final version of the CCU-OHQoL tool (Supplementary files—CCU-OHQoL questionnaire) was administered to a larger sample, to assess construct validity, reliability and acceptability. As per the intended final use, the questionnaire was self-administered.

### Data analysis

There are no definitive criteria for the required sample size in a validation study of this kind. However, previous literature suggests that a sample size of 45 to 50 patients should be sufficient for the proposed analysis [16].

Content validity was assessed through the frequency of endorsement for each item amongst the patients included in the pilot study. For construct validity, the correlation between previously validated global OHQoL items [17] and the score for the items in our questionnaire was analysed using the non-parametric Spearman’s rank correlation coefficient. Furthermore, associations between the CCU-OHQol overall score grouped in categories (i.e. good/fair/poor OHQoL) and global ratings of OHRQoL were tested using Kruskal–Wallis non-parametric test. The reliability of the tool was also assessed: internal consistency was measured using Cronbach’s alpha coefficient and by analysis of the item-total correlation and Cronbach’s Alpha if item deleted. Test–retest reliability was tested via intraclass correlation coefficients.

Differences in self-reported oral health before hospitalisation and at CCU discharge were assessed by Wilcoxon signed rank tests. Overall scores for the CCU-OHQoL were calculated by summing up the response codes since ordinal values were coded for each question as presented in Supplementary files—CCU-OHQoL questionnaire. The distribution of the participants within categories (“Good”, “Fair” or “Poor” OHQoL) was investigated as well as the distribution of responses for all individual items.

We also assessed three summary variables: severity, prevalence and extent; in a similar fashion to that defined by Slade and co-workers [18] and modified to suit the characteristics of our questionnaire:

- *Severity* the sum of ordinal responses
- *Prevalence* the percentage of participants answering one or more item with ordinal values 3 or 4. (i.e. “Very bothered”, “Extremely bothered”, “Dissatisfied”—strongest negative impacts)
- *Extent* the number of items per subject answered with ordinal values 3 or 4

The correlation between the global rating QoL items, the self-reported oral health items and the score for the items in our questionnaire were analysed using the non-parametric Spearman’s rank correlation coefficient. The purpose of assessing these correlations was to explore the relationships between self-reported oral health, OHQoL and QoL overall. Furthermore, the associations between self-reported changes in oral health during CCU stay and overall CCU-OHQoL score were tested using one-way analysis of variance. Analysis was carried out using SPSS Statistics for Windows (Version 22.0, IBM Corp, NY).

## Results

A total of 18 and 45 subjects were recruited for the pilot and validation study, respectively. The demographic characteristics of the study population for both Parts B and C are presented in Table 1.

**Table 1:** Subject characteristics

	Part B: pilot study (*n* = 18)	Part C: validation study (*n* = 45)
Mean age (SD) (years)	55.1 (10.6)	55.8 (17.44)
Gender (*n* (%))		
Women	10 (55.6)	15 (33.3)
Men	8 (44.4)	30 (66.7)
Ethnicity (*n* (%))		
White British	15 (83.3)	27 (60)
White and Black African		2 (4.4)
Asian and Asian British		2 (4.4)
Other White	3 (16.7)	10 (22.2)
White and Black Caribbean		1 (2.2)
Black and Black British		3 (6.7)
Level of care (*n* (%))		
2	13 (72.2)	32 (71.1)
3	5 (27.8)	13 (28.9)
Mean APACHE2 score (SD)	21.6 (10.2)	19.56 (6.72)
Median length of CCU stay	4.5	5
Minimum length of CCU stay	2	2
Maximum length of CCU stay	42	155

### Part A

#### Face and content validity

The expert panel agreed that the tool should include items for the following domains: “satisfaction with oral health”, “functional limitations”, “oral symptoms”, “social impact”, “self-care” and “psychological impact”. Subsequently, a pool of 93 items was scrutinised until consensus was reached for a total of 13 items to be included in the initial tool (Supplementary files—Initial tool, Items 3 to 15). Questions assessing self-reported oral health, three global OHQoL items and a global QoL item were also included.

### Part B

#### Content validity

The analysis of the frequency of endorsement of each item revealed very few items left unanswered (2.9%) and a good distribution of endorsement amongst the available responses for each item. In addition, “floor” and “ceiling” effects were not present when the questionnaire was analysed as a composite score.

During the short interviews, 72.2% (*n *= 13) of the sample reported that the questions were relevant and 12 participants (66.6%) reported that the items related well or very well to their experience during their CCU stay. The addition of questions asking about tooth brushing frequency and bad breath was suggested by five subjects each.

#### Acceptability

The recruitment rate was 47.3% of all subjects asked to participate in the study. The mean time for questionnaire completion was 8 min 33 s (CI 95% 4 min 18 s–12 min 48 s). The majority of the sample (88.9%) thought that the time to complete the questionnaire was reasonable and they would do it again while reporting that the wording of the questions was good (77.7%, *n *= 14) and easy to understand (94.4%, *n *= 17).

#### Modification of the initial pool of domains and items

Following data collection and analysis of the pilot study, the results were reviewed by the expert panel in critical care and oral health that contributed to the development of the initial tool. As a result, two items relating to bad breath and toothbrushing frequency were incorporated.

### Part C

#### Construct validity

The final tool was completed by 45 CCU patients. The correlation coefficient between the CCU-OHQoL score and the overall score of the global items of OHQoL was 0.74 (*p *< 0.001). Furthermore, the CCU-OHQoL score was strongly or moderately correlated with all three global items individually (item 18: 0.70, item 19: 0.61 and item 20: 0.76) being all also statistically significant (*p *< 0.001).

**Fig. 2:**
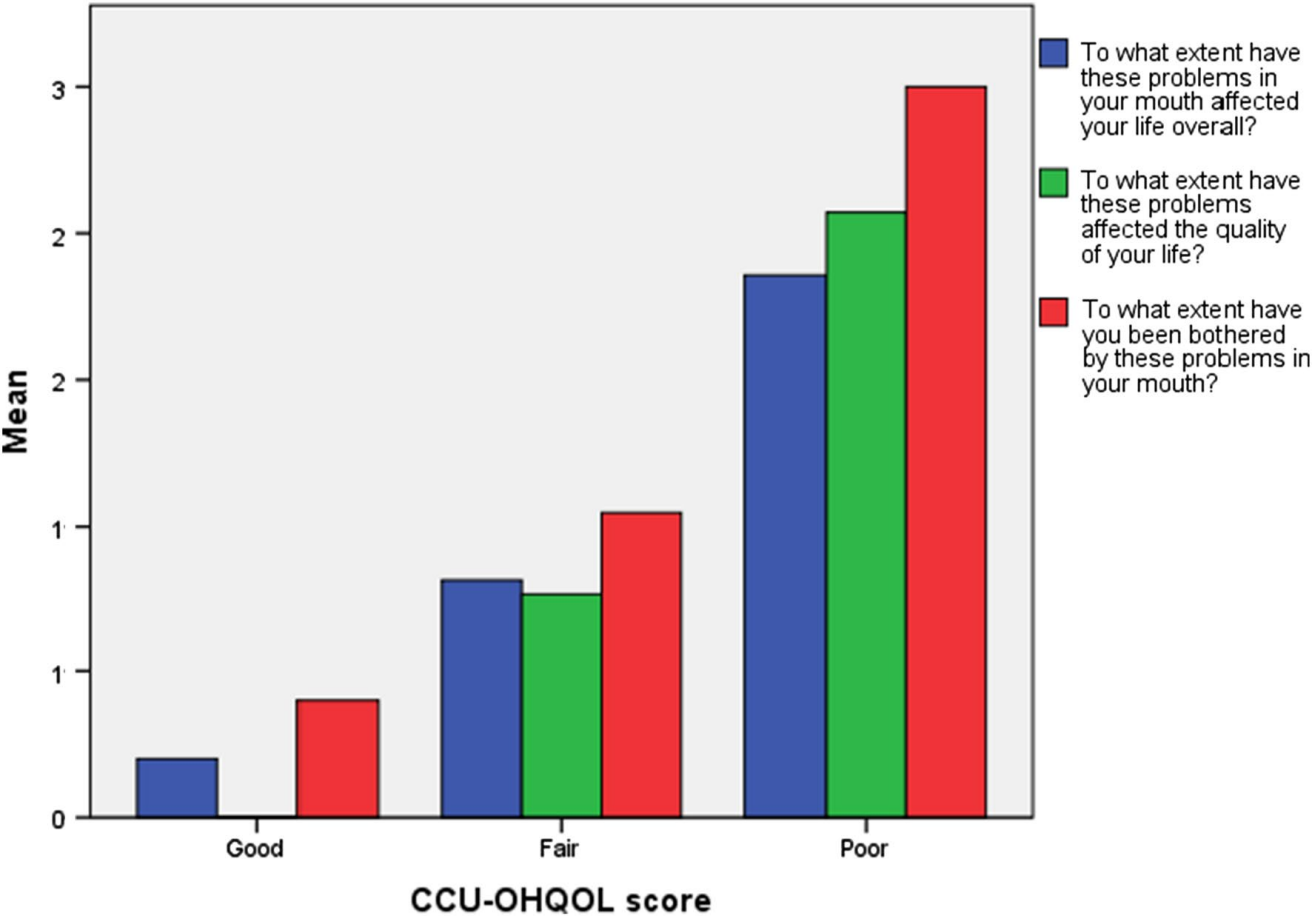
Association between the individual mean ratings for each global OHQoL item and the CCU-OHQoL composite score

There was an association between the categorised version of the CCU-OHQoL tool and the mean rating of the global OHQoL tool which was statistically significant (*p *< 0.001: Kruskal–Wallis non-parametric test). Similar associations and gradients were also observed with each of the global OHQoL items when assessed individually (see Fig. 2).

#### Reliability

Cronbach’s alpha coefficient was 0.72. All of the items of the CCU-OHQoL tool showed an item-total correlation between 0.3 and 0.7 (Supplementary files—item-total correlation) with the exception of questions 3 (0.15) and question 16 (− 0.78). These two questions were also the only items where their deletion resulted in an overall increase in Cronbach’s Alpha (item 3: 0.73; item 16 (0.77). Test–retest reliability was assessed by asking a subset of the subjects (*n *= 5) to complete the questionnaire twice, 5 days apart. The mean intraclass correlation coefficients (ICCs) for the scale overall was 0.65 (95% CI 0.38–0.84).

#### Acceptability

A total of 96 patients were approached to take part in Part C of the study and 45 participants were recruited, translating into a recruitment rate of 46.8%. On average, the questionnaires took the participants 8 min and 53 s (CI 95% 4 min 39 s–13 min 7 s) to complete.

#### Exploratory factor analysis

The dimensionality of the tool was assessed using principal component exploratory factor analysis (EFA). We retained factors with eigenvalues > 1 and rotated them with “Varimax”, which is an orthogonal rotation method. Items were assigned to retain rotated factors when they had a loading of ≥ 0.55 in absolute value. The magnitude of factor loadings, distribution of variance amongst the factors, and the relative correlation of the items with the different factors were assessed.

After Varimax rotation, the first component explained 22% of the variance, and the second principal component added 19.5% to the variance explained (see Table 2). The third, fourth, and fifth components had Eigenvalues of 1.45, 1.28, and 1.13, and explained variances were 12.7%, 10.6%, and 8.8%, respectively. Fourteen out of the 15 items loaded highly on no more than one particular dimension (see Table 3). The four items in the first rotated principal component describe functional limitations and include four out of the five items which were included by our expert panel within this dimension and therefore maintained the name of the original dimension. Items referring to psychosocial impacts (items 14 and 17) were found in the second factor only. In addition, this factor also included items 9 and 10 which were included in the oral symptoms dimension. Item 10 may well have been considered by patients as an indicator of a social interaction and this factor was therefore named “Psychosocial impacts”. The third dimension/factor included the two items measuring symptoms of dry mouth and was therefore named “Xerostomia”. The main item tapping into the 5th factor was question 16. The second highest factor loading (although just short of the cut-off 0.55 value) in this dimension was item 15, which was also the only other item with a factor loading above 0.3 for this component. Since items 15 and 16 were the two items originally included in the dimension “Self-care”, this component retained this name. Finally, the fourth factor referred to questions 3 and 9. Question 3 was included as an item for “Satisfaction with oral health” and seems unrelated to question 9; therefore, it is difficult to discern to which specific dimension these items may be tapping and remained unnamed.

**Table 2:** Exploratory factor analysis: Eigenvalues and % of variance explained before and after rotation

Component	Initial eigenvalues			Extraction sums of squared loadings			Rotation sums of squared loadings		
	Total	% Of variance	Cumulative (%)	Total	% Of variance	Cumulative (%)	Total	% Of variance	Cumulative (%)
1	5.457	36.378	36.378	5.457	36.378	36.378	3.370	22.464	22.464
2	1.783	11.885	48.263	1.783	11.885	48.263	2.922	19.477	41.941
3	1.452	9.679	57.943	1.452	9.679	57.943	1.905	12.699	54.640
4	1.279	8.530	66.472	1.279	8.530	66.472	1.589	10.594	65.234
5	1.136	7.571	74.043	1.136	7.571	74.043	1.321	8.809	74.043

*Extraction method* principal component analysis. *Rotation method* Varimax with Kaiser normalisation

**Table 3:** EFA: rotated component matrix

Components: 1 functional limitations, 2 psychosocial impact, 3 xerostomia, 5 self-care	Component				
	1	2	3	4	5
*Satisfaction with oral health* 3. How dissatisfied or satisfied have you been with the health of your teeth or mouth? *Functional limitation*				0.787	
4. How bothered have you been by having trouble biting or chewing any kinds of food?	0.832				
5. How bothered have you been by your teeth or dentures preventing you from speaking the way you want?	0.725				
6. How difficult did you find it to swallow comfortably?	0.671				
7. How much have you felt that your sense of taste has worsened because of problems with your mouth, teeth, gums or dentures?	0.822				
8. How happy were you with your ability to taste your food? *Oral symptoms*				0.813	
9. How bothered were you by pain in your mouth, teeth or gums?		0.805			
10. How bothered have you been by having to seek help from your nurse or visitors to relieve pain or discomfort from your mouth, teeth or gums?		0.865			
11. How satisfied were you with how moist your mouth feels?			0.899		
12. How bothered have you been about dryness of your mouth?			0.729		
13. How bothered have you been by having bad breath? *Social impact*					
14. How much has the condition of your mouth affected your contacts with members of the hospital staff or visitors (i.e. family and friends)? *Self-care*		0.582			
15. How difficult was for you or the hospital staff to be able to brush your teeth properly because of problems with your mouth, teeth or gums?					0.488*
16. How satisfied were you with how frequently you were able to brush your teeth compared to your home routine? *Psychological impact*					0.891
17. How anxious or self-conscious did you feel because of problems with your mouth, teeth, gums or dentures?		0.683			

Rotation converged in 6 iterations

*Extraction method* principal component analysis, *rotation method* Varimax with Kaiser normalisation

*Below 0.55 cut-off value

#### Patient-reported outcome measures

After their care in CCU, 37.8% reported a deterioration in self-reported oral health which was statistically significant (*p *< 0.03). The mean severity score (CCU-OHQoL overall score) for all participants was 8.96 (95% CI 5.57–12.34) with 31.3% of the sample showing “Poor” OHQoL when assessing their overall score. In terms of prevalence, 42.4% of the participants answered at least two items with an item code of 3 or 4 (stronger negative impacts). Furthermore, the mean extent score was 1.91 (95% CI 1.19–2.62). The distribution of responses to the items of the CCU-OHQoL questionnaire is available in the supplementary files (Supplementary files—frequency of endorsement), while the scores of the global OHQol and QoL items are presented in Table 4. In addition, nearly 40% of the participants (*n *= 18) experienced negative impacts that bothered them at least “somewhat”, 26.7% reported that the negative impacts affected their life overall at least “somewhat” and 31.1% had negative impacts that affected their quality of life at least “somewhat”. Those who reported a deterioration in self-reported oral health showed a higher mean CCU-OHQoL score (12.22, CI 95% 6.85–17.6) compared to those whose self-reported oral health did not change (6.78, CI 95% 2.34–11.22) although this was not statistically significant (*p *= 0.11).

**Table 4:** Scores of global OHQoL and QoL items

Question/item	Not at all *n* (%)	A little *n* (%)	Somewhat *n* (%)	A fair amount *n* (%)	A great deal *n* (%)
To what extent have you been bothered by these problems in your mouth?	12 (26.7)	15 (33.3)	10 (22.2)	6 (13.3)	2 (4.4)
To what extent have these problems in your mouth affected your life overall?	19 (42.2)	14 (31.1)	7 (15.6)	3 (6.7)	2 (4.4)
To what extent have these problems affected the quality of your life?	22 (48.9)	9 (20)	8 (17.8)	4 (8.9)	2 (4.4)

	Very poor *n* (%)	Poor *n* (%)	Fair *n* (%)	Good *n* (%)	Very good *n* (%)
How would you rate the quality of your life?	2 (4.4)	6 (13.3)	8 (17.8)	17 (37.8)	12 (26.7)

## Discussion

### Summary of key findings

The CCU-OHQoL showed clear face and content validity. For construct validity, the tool achieved very strong correlations with global OHQoL (*p* < 0.01) in the expected direction with higher CCU-OHQoL scores indicating worse OHQoL. Furthermore, participants with a “Poor OHQoL” showed greater impacts in their overall life and quality of life. Reliability was adequate with a Cronbach's alpha of 0.72, above the acceptable threshold of 0.7 [19], and adequate test–retest intraclass correlation coefficient of 0.66 (95% CI 0.38–0.84). Therefore, these data suggest that the CCU-OHQoL has appropriate characteristics to be used for the stated purpose.

According to our results, hospitalisation in CCU has a negative effect on the self-perceived oral health of patients. More interestingly, those who reported a deterioration in self-perceived oral health, also showed a higher mean CCU-OHQoL score (poorer OHQoL). Although the correlation marginally failed to show statistical significance (*p* = 0.056), there was a clear trend with nearly a twofold increase in the overall CCU-OHQoL score which may have reached significance in a larger sample. This could be understood as initial evidence to suggest that changes in oral health after hospitalisation, as perceived by the patients, may indeed have an effect on their quality of life.

We also obtained a cross-sectional view of the OHQoL amongst the participants. We use estimates of prevalence, extent and severity as they provide important complimentary information when interpreting OHQoL data [20]. These estimates show significant impacts of oral health on quality of life in our sample. The impacts seem to be more prevalent and intense in the “functional limitations”, “oral symptoms” and “self-care” domains and relatively more modest for “psychosocial impact”. In addition, the overall QoL items revealed that a significant proportion of the sample had impacts that affected their life overall.

### Comparison with other studies

To date, there are no other studies exploring self-reported oral health and OHQoL in the critically ill which precludes comparison of these results. The current results suggest a very strong impact of “xerostomia” in the OHQoL of the critically ill. This is consistent with previous evidence reporting that intubated CCU patients have a significantly reduced salivary flow during hospitalisation [21].

In addition, there are some studies suggesting a deterioration of OHQoL in patients admitted to hospital outside CCUs [22, 23]. Similar to our findings, hospitalisation resulted in worsening of oral health and low OHQoL in other hospitalised populations [22–24]. Yu and co-workers showed that for Chinese geriatric patients, the disruption of the normal daily living routine as a result of hospitalisation translated to a worsening of oral health and low OHQoL [22]. Additionally, Schimmel and co-workers compared the OHQoL of 31 hospitalised stroke patients who presented with hemi-facial and/or limb palsy at the University Hospital of Geneva (Switzerland) to that of 24 subjects with similar age, gender and dental status [23]. The results also indicated a lower OHQoL in the hospitalised population compared with the control group. The OHIP-EDENT [25] mean score was 12.3 ± 17.7 in the control group and 18.8 ± 15.5 in the hospitalised population (*p* = 0.01), with higher scores indicating lower OHQoL. Also in accordance with the previous studies, Cornejo et al. [24] in a cross-sectional study amongst 194 institutionalised elderly in Barcelona (Spain) found that 67% of the participants had poor OHQoL and this was associated with self-reported poor oral health. All in all, it seems that the decline of quality of life due to a worsening of the oral health might be more marked in unwell and hospitalised patients. Extrapolating what we know about the impact of critical care on the HQoL and the impact of poor oral health on the OHQoL of medically compromised patients as discussed above in addition to the results of our study, it could be possible that some of the deterioration in HQoL of the critically ill is due to changes in oral health. In order to improve patient care, we need to know what OHQoL means to patients and what experiences they relate to their stay in CCU. Experiences of critically ill patients are an important aspect of the quality of the care and are essential to guide bedside decisions in the CCU; they should be placed at the centre of public health debate.

### Strengths and limitations

The strengths of the study include the systematic process followed to develop the tool tailored to our target population. The CCU-OHQoL tool is “patient-centred and incorporates aspects of daily living that patients deem to be important” as the patients themselves were involved in the development of the tool [14]. Ideally, participants could have been involved earlier in the process via interviews at the stage of item generation to further limit the risk of critical items being overlooked. The aim was to develop a questionnaire that would not be burdensome on patients or staff to complete. The time for completion of the questionnaire was short and 89% of the patients reported they would be happy to repeat the questionnaire if needed.

However, we recruited a relatively low number of subjects in the final phase of the project and the low recruitment rates may suggest some degree of selection bias; based on the data presented in Table 1, the population in our study seems to be representative of the patients seen at the CCU at UCH. Forty-five patients are at the lower end of what would be considered acceptable [16]. In addition, the sample size for test–retest analysis was also small, precluding meaningful interpretation. Completion of the second questionnaire is logistically difficult since many patients may be discharged from the CCU to their homes or to other hospitals.

In addition, in the initial development and validation of the tool we did not complete an oral examination to assess objectively the oral health of the participants but rather used a previously validated self-reported measure of oral health. Further studies using the CCU-OHQoL and including objective clinical assessments of the oral health of the participants would allow assessment of the responsiveness of the tool and also provide an opportunity to compare the questionnaire against other generic OHQoL tools.

Finally, the cross-sectional analysis of the OHQoL of the participants should be interpreted with caution since the study was designed with the primary aim of developing and validating the CCU-OHQoL tool. Without a control group, the changes might have been due to secular effects not related to CCU. It is not clear what the ideal control group should be. Nonetheless, assessing the oral health and OHQoL of patients admitted to other hospital wards or institutionalised in nursing homes might allow meaningful comparisons even if not representing a true control group [9, 26]. It is also worth noting that it has been proposed that some of the changes in OHQoL and QoL may be due to psychological adjustments such as changes in expectations due to illness which may be particularly relevant in the CCU due to the “response shift” that patients hospitalised in critical care may suffer.

### Implications for research and clinical practice

Further evaluation of the CCU-OHQoL tool is needed to test its psychometric properties and its applicability in other CCU settings. Future studies should provide information regarding test–retest stability and the ability of the tool to discriminate between groups with different levels of oral health assessed by traditional clinical measures. Finally, the responsiveness of the questionnaire could be studied through administration as part of a randomised controlled trial [27].

As perceived by the patients themselves, our results contribute to the body of evidence suggesting a deterioration in oral health following hospitalisation. More importantly, this deterioration was associated with poorer OHQoL indicating that these changes may indeed have an impact in the life and well-being of those hospitalised in a CCU. Since the care received at the CCU would ultimately influence the oral health of the critically ill, this is an extremely important finding encouraging the search of new interventions and care pathways to maintain oral health following admission to a CCU, being this an important public health matter. By including this OHQoL tool in future research, influential data will be provided to decision makers for the development of health promotion and may help to identify a group of patients for whom new care pathways aimed at improving oral health are needed [28]. Simple CCU nurse-led interventions have already been shown to maintain oral health [10] and therefore this is an achievable objective.

## Conclusions

1. This study is the first to develop a tool to measure OHQoL in patients hospitalised in a CCU: the CCU-OHQoL. The new tool has good acceptability, clear face, content and construct validity and satisfactory internal consistency reliability.
2. For a large proportion of the patients, self-rated oral health deteriorated following hospitalisation in a CCU and this deterioration seems to be associated with poorer oral health-related quality of life.
3. Overall, there were substantial negative effects of oral health on the patient’s quality of life during their stay at the CCU with a high prevalence, extent and severity of impacts in the patient’s daily living and quality of life.

## Electronic supplementary material

Below is the link to the electronic supplementary material.

Supplementary material 1 (PDF 79 kb)

Supplementary material 2 (PDF 86 kb)

Supplementary material 3 (PDF 41 kb)

Supplementary material 4 (PDF 116 kb)
