# Supplementary material for: Development of a tool to assess oral health-related quality of life in patients hospitalised in critical care
Source: Qual Life Res. 2019 Oct 26;29(2):559–68. doi: 10.1007/s11136-019-02335-1 (PMC6994456; doi:10.1007/s11136-019-02335-1)
Supplement: Supplementary file 2 — Supplementary material 2 (PDF 86 kb) [file 11136_2019_2335_MOESM2_ESM.pdf]

CCU-OHQoL items: frequency of endorsement.

	Dissatisfied	Somewhat dissatisfied	Neither Satisfied nor dissatisfied	Somewhat satisfied	Satisfied
	n (%)	n (%)	n (%)	n (%)	n (%)
3. How dissatisfied or satisfied have you been with the health of your teeth or mouth?	2 (4.4)	6 (13.3)	22 (48.9)	7 (15.6)	8 (17.8)
	**Not at all**	**Slightly bothered**	**Moderately bothered**	**Very bothered**	**Extremely bothered**
4. How bothered have you been by having trouble biting or chewing any kinds of food?	30 (69.8)	5 (11.6)	4 (9.3)	3 (7.0)	1 (2.2)
5. How bothered have you been by your teeth or dentures preventing you from speaking the way you want?	35 (77.8)	5 (11.1)	2 (4.4)	2 (4.4)	1 (2.2)
	**Not at all**	**Slightly difficult**	**Moderately difficult**	**Very difficult**	**Extremely difficult**
6. How difficult did you find it to swallow comfortably?	23 (51.1)	11 (24.4)	3 (6.7)	4 (8.9)	4 (8.9)
	**Not at all**	**A little**	**Somewhat**	**A fair amount**	**A great deal**
7. How much have you felt that your sense of taste has worsened because of problems with your mouth, teeth, gums or dentures?	22 (50)	9 (20.5)	11 (25)		2(4.5)
	**Unhappy**	**Somewhat unhappy**	**Neither happy nor unhappy**	**Somewhat happy**	**Happy**
8. How happy were you with your ability to taste your food?	7 (16.3)	3 (7.0)	13 (30.2)	4 (9.3)	16 (37.2)
	**Not at all**	**Slightly bothered**	**Moderately bothered**	**Very bothered**	**Extremely bothered**
9. How bothered were you by pain in your mouth, teeth or gums?	31 (68.9)	4 (8.9)	3 (6.7)	4 (8.9)	3 (6.7)
10. How bothered have you been by having to seek help from your nurse or visitors to relieve pain or discomfort from your mouth, teeth or gums?	39 (86.7)	1 (2.2)	1 (2.2)	2 (4.4)	2 (4.4)
	**Dissatisfied**	**Somewhat dissatisfied**	**Neither Satisfied nor dissatisfied**	**Somewhat satisfied**	**Satisfied**
11. How satisfied were you with how moist your mouth feels?	10 (22.3)	13 (28.9)	10 (22,2)	5 (11.1)	7 (15.6)
	**Not at all**	**Slightly bothered**	**Moderately bothered**	**Very bothered**	**Extremely bothered**
12. How bothered have you been about dryness of your mouth?	6 (13.3)	13 (28.9)	11 (24.4)	7 (15.6)	8 (17.8)
13. How bothered have you been by having bad breath?	16(35.6)	15 (33.3)	8 (17.8)	4 (8.9)	2 (4.4)
	**Not at all**	**A little**	**Somewhat**	**A fair amount**	**A great deal**
14. How much has the condition of your mouth affected your contacts with members of the hospital staff or visitors (i.e. family and friends)?	27 (60)	8 (17.8)	7 (15.6)	1 (2.2)	2 (4.4)
	**Not at all**	**Slightly difficult**	**Moderately difficult**	**Very difficult**	**Extremely difficult**
15. How difficult it was for you or the hospital staff to be able to brush your teeth properly because of problems with your mouth, teeth or gums?	24 (54.4)	9 (20.5)	6 (13.6)	4 (9.1)	1 (2.3)
	**Dissatisfied**	**Somewhat dissatisfied**	**Neither Satisfied nor dissatisfied**	**Somewhat satisfied**	**Satisfied**
16. How satisfied were you with how frequently you were able to brush your teeth compared to your home routine?	7 (15.6)	14 (31.1)	11 (24.4)	5 (11.1)	8 (17.8)
	**Not at all**	**Slightly anxious or self-conscious**	**Moderately anxious …**	**Very anxious or self-conscious**	**Extremely anxious…**

17. How anxious or self-conscious did you feel because of problems with your mouth, teeth, gums or dentures?	22 (48.9)	15 (33.3)	5 (11.1)	1 (2.2)	2 (4.4)
