# Supplementary material for: Development of a tool to assess oral health-related quality of life in patients hospitalised in critical care
Source: Qual Life Res. 2019 Oct 26;29(2):559–68. doi: 10.1007/s11136-019-02335-1 (PMC6994456; doi:10.1007/s11136-019-02335-1)
Supplement: Supplementary file 3 — Supplementary material 3 (PDF 41 kb) [file 11136_2019_2335_MOESM3_ESM.pdf]

Item-total correlation and Cronbach´s alpha if item deleted.

	Corrected Item-Total Correlation	Cronbach's Alpha if Item Deleted
**Satisfaction with oral health**<br>3. How dissatisfied or satisfied have you been with the health of your teeth or mouth?	.151	.733
**Functional limitation**<br>4. How bothered have you been by having trouble biting or chewing any kinds of food?	.459	.702
5. How bothered have you been by your teeth or dentures preventing you from speaking the way you want?	.453	.705
6. How difficult did you find it to swallow comfortably?	.534	.690
7. How much have you felt that your sense of taste has worsened because of problems with your mouth, teeth, gums or dentures?	.541	.696
8. How happy were you with your ability to taste your food?	.340	.720
**Oral Symptoms**<br>9. How bothered were you by pain in your mouth, teeth or gums?	.450	.698
10. How bothered have you been by having to seek help from your nurse or visitors to relieve pain or discomfort from your mouth, teeth or gums?	.452	.702
11. How satisfied were you with how moist your mouth feels?	.347	.714
12. How bothered have you been about dryness of your mouth?	.597	.685
13. How bothered have you been by having bad breath?	.379	.707
**Social impact**<br>14. How much has the condition of your mouth affected your contacts with members of the hospital staff or visitors (i.e. family and friends)?	.324	.713
**Self-care**<br>15. How difficult it was for you or the hospital staff to be able to brush your teeth properly because of problems with your mouth, teeth or gums?	.687	.684
16. How satisfied were you with how frequently you were able to brush your teeth compared to your home routine?	-.078	.779
**Psychological Impact**<br>17. How anxious or self-conscious did you feel because of problems with your mouth, teeth, gums or dentures?	.566	.698
