# Supplementary material for: Development of a tool to assess oral health-related quality of life in patients hospitalised in critical care
Source: Qual Life Res. 2019 Oct 26;29(2):559–68. doi: 10.1007/s11136-019-02335-1 (PMC6994456; doi:10.1007/s11136-019-02335-1)
Supplement: Supplementary file 4 — Supplementary material 4 (PDF 116 kb) [file 11136_2019_2335_MOESM4_ESM.pdf]

Final CCU-OHQoL tool with scale responses in ordinal values (scores)

At the end, you will be asked to record how long it took to complete the questionnaire.
Please write down what time is it now, before you start filling in the questionnaire.

TIME	:

We would like you to answer the following questions. Please circle one answer per question.

1. How would you describe the health of your teeth or mouth before being in hospital?

	ANSWER
Very poor	1
Poor	2
Fair	3
Good	4
Very good	5

2. How would you describe the health of your teeth or mouth now?

	ANSWER
Very poor	1
Poor	2
Fair	3
Good	4
Very good	5

During your stay at the Critical Care Unit:

3. How dissatisfied or satisfied have you been with the health of your teeth or mouth?

	ANSWER	SCORE
Dissatisfied	1	4
Somewhat dissatisfied	2	2
Neither satisfied nor dissatisfied	3	0
Somewhat satisfied	4	-2
Satisfied	5	-4

4. How bothered have you been by having trouble biting or chewing any kinds of food?

	ANSWER	SCORE
Not at all	1	0
Slightly bothered	2	1
Moderately bothered	3	2
Very bothered	4	3
Extremely bothered	5	4

5. How bothered have you been by your teeth or dentures preventing you from speaking the way you want?

	ANSWER	SCORE
Not at all	1	0
Slightly bothered	2	1
Moderately bothered	3	2
Very bothered	4	3
Extremely bothered	5	4

6. How difficult did you find it to swallow comfortably?

	ANSWER	SCORE
Not at all	1	0
Slightly difficult	2	1
Moderately difficult	3	2
Very difficult	4	3
Extremely difficult	5	4

7. How much have you felt that your sense of taste has worsened because of problems with your mouth, teeth, gums or dentures?

	ANSWER	SCORE
Not at all	1	0
A little	2	1
Somewhat	3	2
A fair amount	4	3
A great deal	5	4

8. How happy were you with your ability to taste your food?

	ANSWER	SCORE
Unhappy	1	4
Somewhat unhappy	2	2
Neither unhappy nor happy	3	0
Somewhat happy	4	-2
Happy	5	-4

9. How bothered were you by pain in your mouth, teeth or gums?

	ANSWER	SCORE
Not at all	1	0
Slightly bothered	2	1
Moderately bothered	3	2
Very bothered	4	3
Extremely bothered	5	4

10. How bothered have you been by having to seek help from your nurse or visitors to relieve pain or discomfort from your mouth, teeth or gums?

	ANSWER	SCORE
Not at all	1	0
Slightly bothered	2	1
Moderately bothered	3	2
Very bothered	4	3
Extremely bothered	5	4

11. How satisfied were you with how moist your mouth feels?

	ANSWER	SCORE
Dissatisfied	1	4
Somewhat dissatisfied	2	2
Neither satisfied nor dissatisfied	3	0
Somewhat satisfied	4	-2
Satisfied	5	-4

12. How bothered have you been about dryness of your mouth?

	ANSWER	SCORE
Not at all	1	0
Slightly bothered	2	1
Moderately bothered	3	2
Very bothered	4	3
Extremely bothered	5	4

13. How bothered have you been by having bad breath?

	ANSWER	SCORE
Not at all	1	0
Slightly bothered	2	1
Moderately bothered	3	2
Very bothered	4	3
Extremely bothered	5	4

14. How much has the condition of your mouth affected your contacts with members of the hospital staff or visitors (i.e. family and friends)?

	answer	score
A great deal	5	4
A fair amount	4	3
Somewhat	3	2
A little	2	1
Not at all	1	0

15. How difficult it was for you or the hospital staff to be able to brush your teeth properly because of problems with your mouth, teeth or gums?

	answer	score
Not at all	1	0
Slightly difficult	2	1
Moderately difficult	3	2
Very difficult	4	3
Extremely difficult	5	4

16. How satisfied were you with how frequently you were able to brush your teeth compared to your home routine?

	answer	score
Dissatisfied	1	4
Somewhat dissatisfied	2	2
Neither satisfied nor dissatisfied	3	0
Somewhat satisfied	4	-2
Satisfied	5	-4

17. How anxious or self-conscious did you feel because of problems with your mouth, teeth, gums or dentures?

	answer	score
Not at all	1	0
Slightly anxious or self-conscious	2	1
Moderately anxious or self-conscious	3	2
Very anxious or self-conscious	4	3
Extremely anxious or self-conscious	5	4

Throughout this questionnaire, we have asked you about a number of different problems that you may have experienced because of the condition of your mouth and teeth.

18. To what extent have you been bothered by these problems in your mouth?

	answer
Not at all	1
A little	2
Somewhat	3
A fair amount	4
A great deal	5

19. To what extent have these problems in your mouth affected your life overall?

	answer
Not at all	1
A little	2
Somewhat	3
A fair amount	4
A great deal	5

20. To what extent have these problems affected the quality of your life?

	answer
Not at all	1
A little	2
Somewhat	3
A fair amount	4
A great deal	5

21. How would you rate the quality of your life?

	answer
Very good	5
Good	4
Fair	3
Poor	2
Very poor	1

Please write how much time, in minutes, you took to complete the questionnaire

		MINUTES

THANK YOU VERY MUCH FOR COMPLETING THIS QUESTIONNAIRE

The overall CCU-OHQoL was categorised as follows: “Good OHQoL” for scores between -16 and 0, “Fair OHQoL” for scores between 1and 16, “Poor OHQoL” for scores between 17 to 32, “ Very poor OHQoL” for those with scores over 32. These arbitrary cut-off points were determined according to the relative intensity and/or frequency of reported impacts needed to obtain the scores associated with the different categories
